# A model for predicting degree of malignancy in patients with intraductal papillary mucinous neoplasm

**DOI:** 10.3389/fonc.2023.1087852

**Published:** 2023-01-24

**Authors:** Xiangyi He, Rong Fan, Jing Sun, Yanhao Ren, Xuesong Zhao, Weiwei Rui, Yaozong Yuan, Duowu Zou

**Affiliations:** ^1^ Department of Gastroenterology, Ruijin Hospital, Shanghai Jiao Tong University School of Medicine, Shanghai, China; ^2^ School of Mathematical Sciences, Fudan University, Shanghai, China; ^3^ Departments of Radiology, Ruijin Hospital, Shanghai Jiao Tong University School of Medicine, Shanghai, China; ^4^ Departments of Pathology, Ruijin Hospital, Shanghai Jiao Tong University School of Medicine, Shanghai, China

**Keywords:** high grade dysplasia, PT1a, intraductal papillary mucinous neoplasm, model, pancreas

## Abstract

**Background/Objectives:**

There is no predictive model available to address early stage malignant intraductal papillary mucinous neoplasm (IPMN) including high grade dysplasia (HGD) and pT1a (invasive component≤0.5 cm). The aim of this study was to establish an objective and sufficient model to predict the degree of malignancy in patients with IPMN, which can be easily applied in daily practice and adopted for any type of lesion.

**Methods:**

A retrospective cohort study of 309 patients who underwent surgical resection for IPMN was performed. Members of the cohort were randomly allocated to the training or testing set. A detection tree model and random forest model were used for a 3-class classification to distinguish low grade dysplasia (LGD), HGD/pT1a IPMN, and invasive intraductal papillary mucinous cancer (I-IPMC) beyond pT1a.

**Results:**

Of the 309 patients, 54 (17.4%) had early stage malignancy (19 HGD, 35 pT1a), 49 (15.9%) had I-IPMC beyond pT1a, and 206 (66.7%) had LGD IPMN. We proposed a 3-class classification model using a random forest algorithm, and the model had an accuracy of 99.5% with the training set, and displayed an accuracy of 96.0% with the testing set. We used SHAP for interpretation of the model and showed the top five factors (mural nodule size, main pancreatic duct diameter, CA19-9 levels, lesion edge and common bile duct dilation) were most likely to influence the 3-class classification results in terms of interpretation of the random forest model.

**Conclusions:**

This predictive model will help assess an individual’s risk for different stages of IPMN malignancy and may help identify patients with IPMN who require surgery.

## Introduction

Intraductal papillary mucinous neoplasms (IPMNs) are precursor lesions of pancreatic adenocarcinoma (1). Classification of low grade dysplasia (LGD), high grade dysplasia (HGD), and invasive intraductal papillary mucinous carcinoma (I-IPMC) is based on the degree of cyto-architectural dysplasia in accordance with the World Health Organization (WHO) classification system published in 2019 (2). Several reports have shown that I-IPMC has characteristics similar to pancreatic ductal adenocarcinoma (PDAC), including potential lymph node (LN) or distant metastasis, postoperative recurrence, and poor survival (3–9). Depending on the grade of IPMN during the progression from noninvasive IPMN to invasive IPMC, the choice of treatment varies from a conservative approach to radical pancreatectomy with LN dissection (10). Therefore, it is important to determine the grade of IPMN accurately to optimize the therapy and follow-up strategy. Preoperative distinction of IPMN grade is not easy, even when multiple modalities are used (10–17). Several guidelines propose radiological and clinical criteria to assess the risk of HGD and cancer in patients affected by IPMN (18–21), such as the International Consensus Guidelines (ICG) published in 2012, in which predictors of malignancy were categorized as high risk stigmata and worrisome features (18). However, the predicting model still does not identify the degree of malignancy in patients with IPMN.

Although HGD is not malignant, HGD-IPMN has been shown to exhibit an increased risk of subsequent development of pancreatic cancer in a few studies. Therefore, HGD and I-IPMC were studied together in various guidelines (9, 18–24). However, the median overall survival for patients who underwent pancreatic resection for IPMN with HGD was similar to the survival of patients with LGD, and superior to that of patients with IPMN-associated PDAC (22). Thus, early recognition of HGD is important to improve prognosis of the disease.

The recent consensus is that the malignant potential of IPMN is dependent on the presence and extent of invasive cancer (24–30). However, I-IPMC categorized by the Armed Forces Institute of Pathology (AFIP) and WHO classifications covers cancer with variable biological behaviors, with the 5-year survival rate varying substantially between 36% and 90% (2, 24–30). This may be due to the heterogeneity of I-IPMC, including biological behavior and an invasive component of various sizes. Invasion of ≤ 0.5 cm presents with excellent prognosis whereas the prognosis for IPMN at an advanced stage of invasion is as poor as that of conventional PDAC (2). In addition, no LN metastasis was observed in patients with early stage invasion ≤ 5 mm (31, 32). Thus, it would be more appropriate to stage invasive carcinomas with conventional staging protocols according to WHO (2019) and then further substage the pT1 category (early invasion ≤ 2 cm) into pT1a for those with an invasive component ≤ 5 mm (2). This is important for the early detection of pancreatic cancer.

HGD and pT1a are considered as the early stage of malignant IPMN. The ability to distinguish HGD or pT1a IPMN from LGD IPMN will help physicians treat IPMN patients in which it is difficult to identify by clinical and radiological characteristics, allowing for clear surgical indications and the ability to cure pancreatic cancer at an embryonic stage. Many reports have attempted to identify predictive factors for HGD and I-IPMC that might influence the management of IPMN patients by logistic regression analysis and nomogram (10, 16, 17, 31, 32). However, there have been no reports of a subdivided model to predict HGD/pT1a in patients with IPMN that could be widely applied in clinical practice. Therefore, it is necessary to find a simple and objective method to discriminate LGD or HGD/pT1a IPMN and advanced IPMC accurately based on clinical and radiological data.

Decision tree classification algorithms are an inductive learning method that can produce a tree-type classification model from given training samples. Each non-leaf node in the tree shows which feature is used to judge the category, and each leaf node represents the final category. The root node to each leaf node forms a path for classification.

Model interpretation is often essential to illustrate a machine learning model, especially in medical research. The factors which have strong significance in a classification or regression model can help medical workers attach importance to certain factors. An intuitive interpretation of a model can be easily understood. Scott ML et al. developed the method of calculating the *SHAP value* for interpretation of these models (33). The significance is illustrated by the absolute SHAP value; larger values have stronger significance.

Ensemble learning is a common method in machine learning. While learning, multiple models or classifiers are produced independently and ensembled into a final prediction model. The ensemble model usually performs better than any independent prediction model. The random forest algorithm is a combination of bagging and decision tree. Each tree is produced by part of the training sample and features. The final random forest model ensembles the decision trees by voting. Therefore, the random forest model has a higher accuracy.

## Methods

### Patients

Between December 2008 and October 2018, 372 consecutive patients underwent surgical resection for pathologically confirmed IPMN of the pancreas at Ruijin Hospital, Shanghai Jiaotong University School of Medicine were included. A total of 63 patients were excluded from the study because they had a histological diagnosis concomitant with PDAC (n = 41) or some essential preoperative examinations were not performed before surgery (n = 22). Finally, a total of 309 patients were included in the study. The patients’ demographic information, clinicopathological features, and imaging findings were retrieved and systematically entered into a Microsoft Excel spreadsheet. The study was approved by the ethics committee of Ruijin Hospital, Shanghai Jiaotong University School of Medicine.

### Preoperative examinations

**Figure 1:**
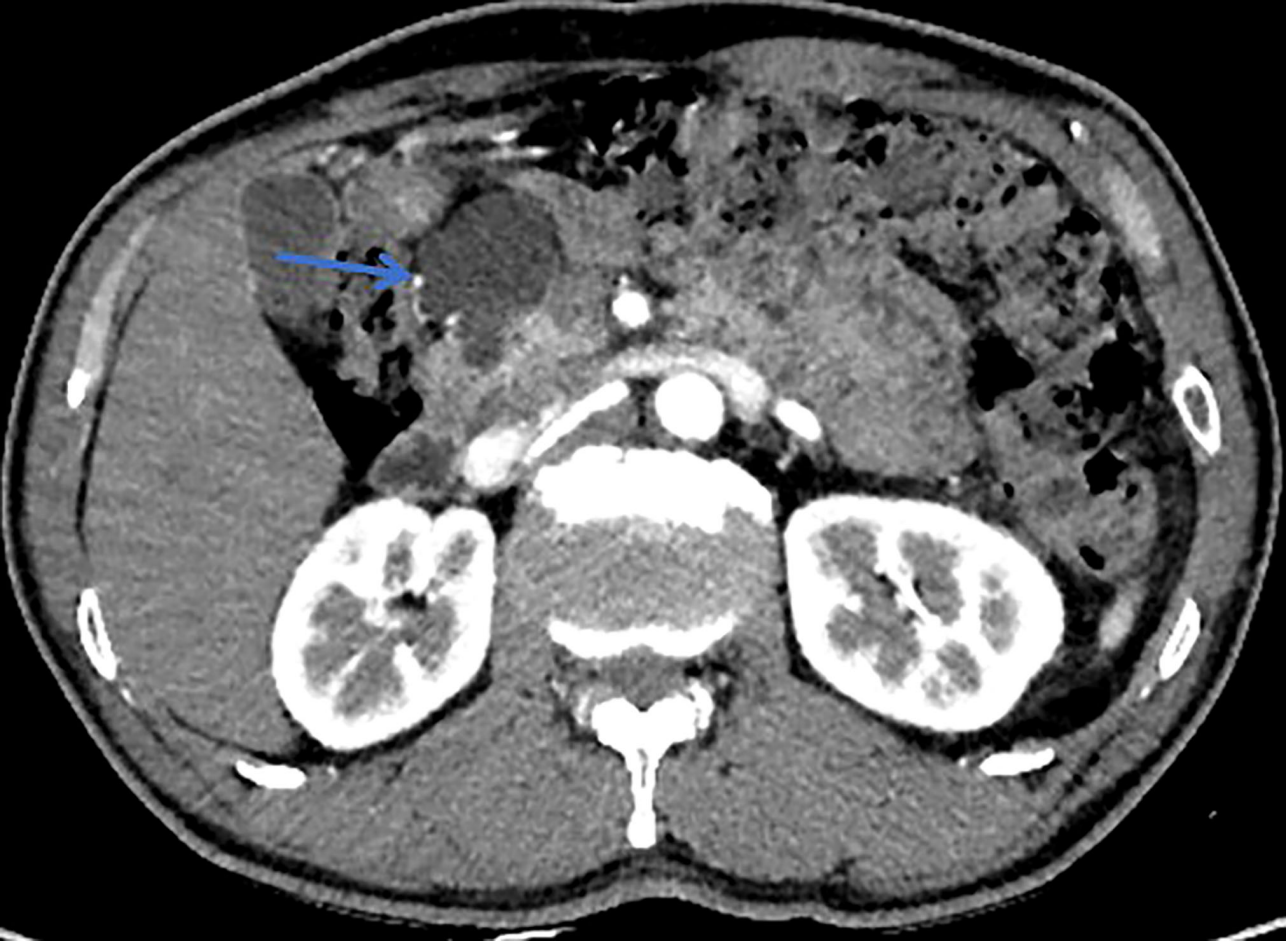
This patient was histologically diagnosed with BD-IPMN. CT with sharp margins of a cystic lesion (blue arrow) in the head of the pancreas.

**Figure 2:**
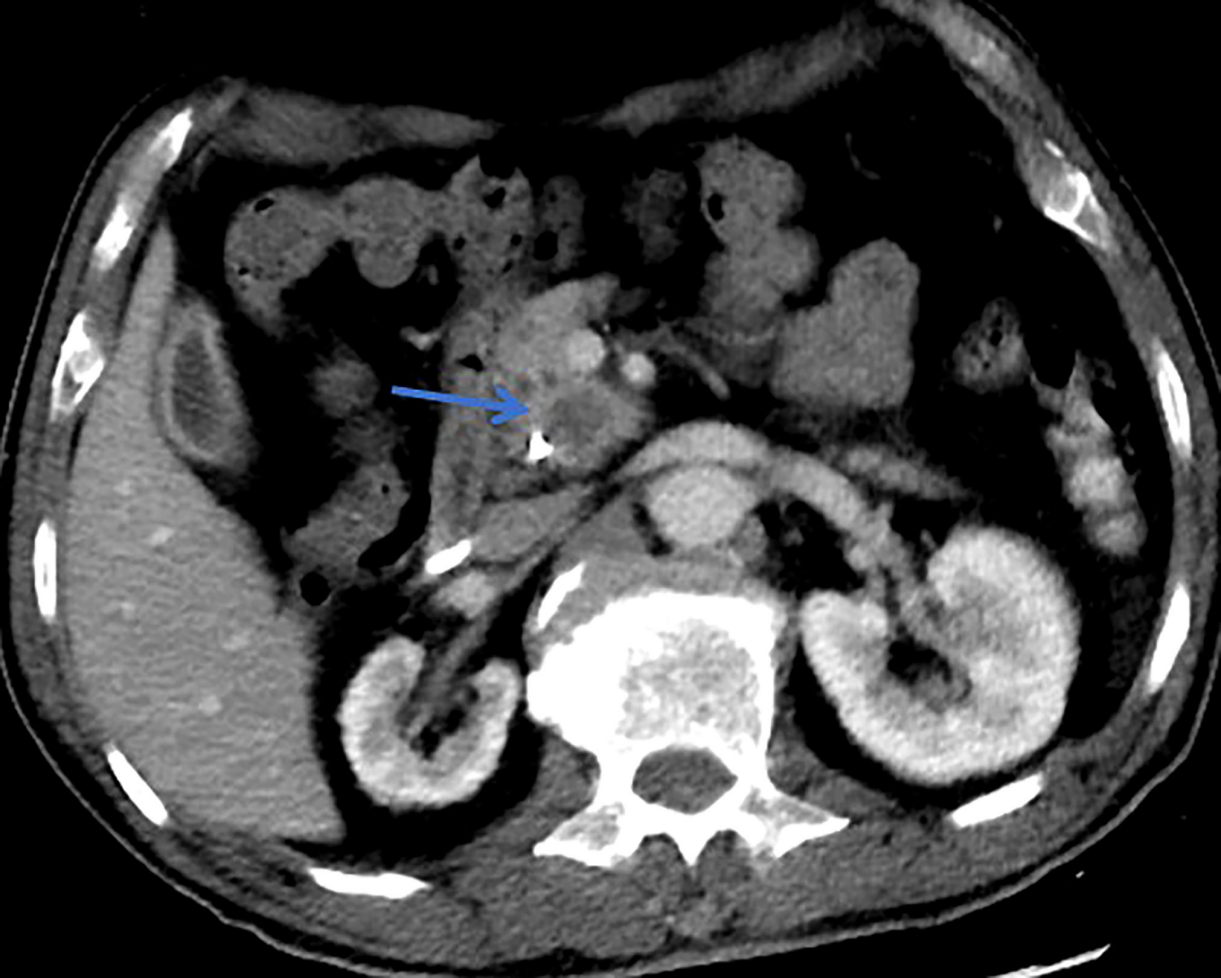
This patient was histologically diagnosed with malignant MT-IPMN. CT with unsharp margins of a cystic lesion (blue arrow) in the uncinate process of the pancreas.

All patients underwent clinical evaluation, laboratory testing, including serum tumor marker levels (carcinoembryonic antigen [CEA] and carbohydrate antigen 19-9 [CA19-9], carbohydrate antigen 125 [CA125], and alpha-fetoprotein [AFP]), pancreatic magnetic resonance imaging (MRI)/MRCP, and computed tomography (CT) before surgery. The morphological type, maximum branch duct cyst size, main pancreatic duct (MPD) diameter, mural nodule size, and internal septations were determined by pancreatic MRI/MRCP. In addition, among the patients who had multifocal pancreatic cystic lesions (two or more), only the largest cyst was measured according to the methods in this study. When there is suspicion of malignant IPMN, assessment of vascular involvement, peritoneal, or metastatic disease were determined by CT. Lesion edge is defined as the radiographic boundary between the lesion and surrounding pancreas whether the edge is clear (**Figures 1**, **2**) (34). Alternative imaging modalities such as endoscopic ultrasound(EUS) and endoscopic retrograde cholangiopancreatography (ERCP) are increasingly being used in the diagnosis of IPMN, but these adjunct diagnostic techniques were not included in this cohort, as data were only available for some of the patients. If surgery is considered, endoscopic ultrasonography-guided fine needle aspiration and biopsy are not routinely performed because of concerns about the possibility of seeding tumor cells along the needle track and high risk of adverse events (intracystic bleeding, acute pancreatitis , fever/infection, et al) (35–38).

Based on preoperative imaging analyses, we classified tumors as main duct (MD), branch duct (BD), or mixed type (MT) IPMN. MD-IPMN was defined as segmental or diffuse dilation of the MPD (> 5 mm). Branch duct IPMN was defined as pancreatic cysts of > 5 mm in diameter that communicate with the MPD, and MT-IPMN patients meet the criteria for both MD-IPMN and BD-IPMN (18). The locations of lesions, according to anatomical characteristics, included the pancreatic head and/or uncinate process, pancreatic neck, pancreatic body, pancreatic tail, and diffuse (when the lesions involved more than one segment of the anatomical location) (39). Common bile duct (CBD) dilatation was defined as dilation greater than 7 mm in patients younger than 60 years, 9 mm in patients older than 60 years, and 10 mm in patients who had undergone a cholecystectomy, with the exception of CBD stones (39, 40).

### Surgical indications

Surgery was performed in the patients with IPMN who met at least one of the following criteria: (1) presence of symptom (jaundice, abdominal pain, back pain, weight loss); (2) MD-IPMN (18, 19); (3) MT-IPMN with a MPD diameter of > 7 mm (41); (4) gradual increase in the BD cyst size (total growth ≥10 mm) during follow-up (42); (5) BD or MT IPMN with contrast-enhancing mural nodules size greater than 5 mm (43); and (6) histologically proven invasive IPMN based on EUS-guided biopsy.

### Pathological diagnosis

Pancreatic specimens were serially sectioned at 5-mm intervals, and whole slides were reviewed and graded by a specialized pathologist blinded to clinical outcomes. The grades were LGD, HGD, or I-IPMC, based on the degree of cyto-architectural dysplasia in accordance with the WHO classification system (2). When more than one pathologic type was present in the same patient, the highest degree of dysplasia was recorded. I-IPMC was defined as the presence of a continuous invasive component from HGD in pathological findings to distinguish it from PDAC concomitant with IPMN. I-IPMC tumors were staged according to the Tumor, Node, and Metastasis Classification of Malignant Tumors published by the International Union against Cancer (44), and thereafter the substage of the T1 category of I-IPMC was classified as T1a (invasive component ≤ 5 mm), T1b (invasive component > 5 mm and ≤ 10 mm), or T1c (invasive component > 10 mm and ≤ 20 mm), according to the revised ICG in 2012 and the 2019 WHO classification system (2, 18). The type of I-IPMC was classified as tubular or colloid based on differentiation of the invasive components (18).

### Follow-up

In general, patients with I-IPMC and non-invasive IPMN were followed for recurrence or progression every 3–6 or 6–12 months, respectively, with imaging studies that included at least one of the following: CT scan, MRI, or EUS. Survival time was calculated from the time of surgical resection to death, or the end of follow-up (December 2018), whichever came first. Censoring occurred for patients who were still alive or who died as a result of other reasons at the last follow-up.

### Statistical analysis

To perform model development and testing independently, the cohort was randomly divided into a model development (MD) set and a test set (2:1) in each random seed. Categorical variables are expressed in frequencies and percentages (%) and comparison of ratios was tested by chi-square test. Continuous variables were expressed as mean ± standard deviation (SD) or median and range depending on data contribution. Continuous variables were compared using the Mann-Whitney U-test or paired Student *t-*test. Postoperative overall survival rates were calculated using the Kaplan–Meier method. Bonferroni’s correction was used for pairwise comparisons.

For the tree-type model in our study, we calculate the SHAP value of a trained model given certain pairs of input and output. In particular, the categorical features are all transferred into one hot vector and are concatenated with numerical features. To show the components of each one hot vector, we add numbers at the end of the features for each category. We use the Python package SHAP for calculation, and show the top factors that have the largest absolute SHAP value. Python 3.7 and the function TreeExplainer in SHAP 0.38.1 were used.

## Results

### Patient characteristics

A total of 372 patients underwent pancreatic surgical resection for IPMN. Sixty-three patients were excluded from the study because they had a histological diagnosis concomitant with PDAC (n = 41) or some essential preoperative examinations were not performed before surgery (n = 22). Finally, a total of 309 patients were included in the study. Demographic information, clinicopathological features, and imaging findings of the 309 patients are shown in **Table 1**. No one patient had undergone neoadjuvant chemotherapy prior to operation. Their median (range) age was 62 (27–84) years, and 170 patients (55.0%) were male. With regard to radiographic morphological types, 167 patients (54.0%) had BD-IPMN, 38 (12.3%) had MD-IPMN, and 104 (33.7%) had MT-IPMN. Total pancreatectomy was performed in 16 (5.2%) patients, pancreatic duodenectomy in 167 (54.0%) patients, distal pancreatectomy in 75 (24.3%) patients, and central pancreatectomy in 51 (16.5%) patients. Final pathological diagnosis showed that 206 (66.7%) patients had LGD, 19 (6.1%) patients had HGD, 35 (11.3%) patients had pT1a IPMC, and 49 (15.9%) patients had I-IPMC beyond pT1a. Among 84 patients with I-IPMC, 18 (21.4%) patients had LN metastasis. No LN metastasis was observed in the pT1a IPMC group.

**Table 1:** Clinical characteristics of patients between the training set and testing set.

	Total	Training set	Testing set		
	n=309	n=210	n=99	X2	P value
Sex				0.129	0.806
Male	170 (55%)	117 (55.7%)	53 (53.5%)		
Female	139 (45%)	93 (44.3%)	46 (46.5%)		
Age (mean±SD)	61.80± 8.64 (84,27)	61.37±8.35 (84,27)	62.72± 9.20 (81,30)		0.202
Tumor location				5.374	0.071
Head of pancreas	164 (53.1%)	118 (56.2%)	46 (46.5%)		
Body and tail of pancreas	97 (31.4%)	66 (31.4%)	31 (31.3%)		
Grade of differentiation	48 (15.5%)	26 (12.4%)	22 (22.2%)		
Pathological diagnosis					
IPMN and Low-grade	206 (66.7%)	139 (66.2%)	67 (67.7%)		
High-grade dysplasia	19 (6.1%)	13 (6.2%)	6 (6.1%)		
pT1a IPMC	35 (11.3%)	24 (11.4%)	11 (11.1%)		
Tubular	26	17	9		
Colloid	9	7	2		
Invasion beyond pT1a IPMC	49 (15.9%)	34 (16.2%)	15 (15.2%)		
Tubular	38	25	13		
Colloid	11	9	2		
T stage ^a^					
T1a	35 (41.7%)	24 (41.4%)	11 (42.3%)		
T1b	0 (0%)	0 (0%)	0 (0%)		
T1c	9 (10.7%)	4 (6.9%)	5 (19.2%)		
T2	24 (28.6%)	19 (32.8%)	5 (19.2%)		
T3	16 (19.0%)	11 (19.0%)	5 (19.2%)		
T4	0 (0%)	0 (0%)	0 (0%)		
N stage, N1^b^	18 (21.4%)	12 (20.7%)	6 (23.1%)		
M stage, M1	0 (0%)	0 (0%)	0 (0%)		
UICC stage ^c^					
IA	42 (50%)	27 (46.6%)	15 (57.7%)		
IB	12 (14.3%)	11 (19.0%)	1 (3.8%)		
IIA	12 (14.3%)	8 (13.8%)	4 (15.4%)		
IIB	18 (21.4%)	12 (20.7%)	6 (23.1%)		
III	0	0	0		
IV	0	0	0		
Morphological types on imaging findings				0.415	0.855
Branch duct	167 (54%)	111 (52.9%)	56 (56.6%)		
Main duct	38 (12.3%)	27 (12.9%)	11 (11.1%)		
Mixed	104 (33.7%)	72 (34.3%)	32 (32.3%)		
Surgical procedures				4.477	0.215
Total pancreatectomy	16 (5.2%)	8 (3.8%)	8 (8.1%)		
Pancreaticoduodenectomy	167 (54%)	117 (55.7%)	50 (50.5%)		
Distal pancreatectomy	75 (24.3%)	54 (25.7%)	21 (21.2%)		
Central pancreatectomy	51 (16.5%)	31 (14.8%)	20 (20.2%)		

a T stage indicates the depth of the invasive component. T1a was defined as a depth of 5 mm; T1b as > 5 mm and <10 mm; and T1c as ≥ 10 mm and ≤ 20 mm.

b No lymph node metastasis in the pT1a IPMC group.

c The pathological stage was determined by the 7^th^ edition of the Tumor, Node, and Metastasis.

### Training and testing subgroups

The training and testing sets contained 210 (68%) and 99 (32%) patients, respectively. No clinical or pathological variables were statistically different between the training and test sets, suggesting similarity in the cohorts. Complete demographic and pathological results stratified by training and testing subgroups can be found in **Table 1**.

### Postoperative survival in the training subgroup

**Figure 3:**
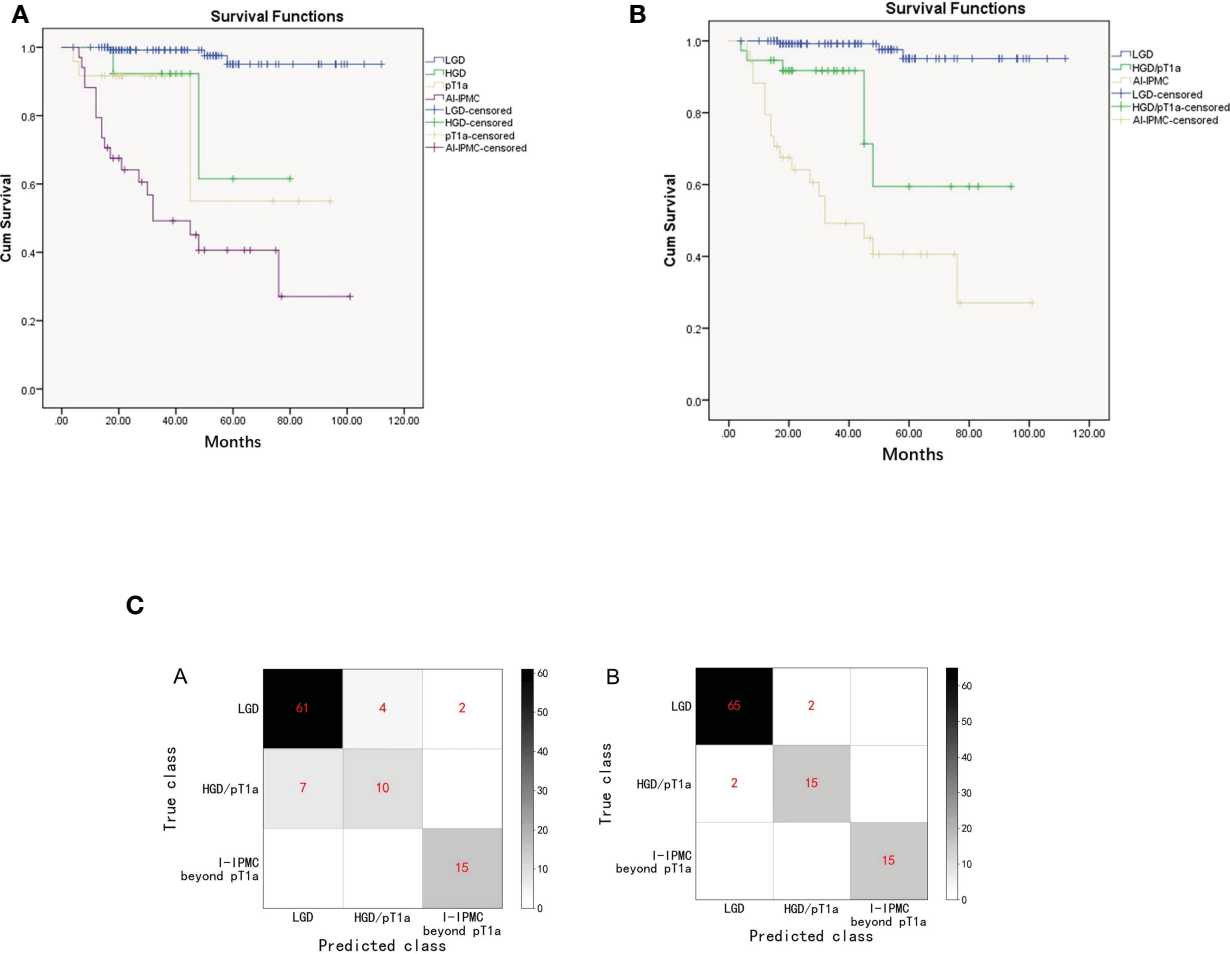
**(A)**. Kaplan–Meier survival estimates of patients with LGD, HGD, pT1a, or I-IPMC beyond the pT1a group undergoing surgical resection of IPMN (Graph 1). **(B)**. Kaplan–Meier survival estimates of patients with LGD, HGD/pT1a, or I-IPMC beyond the pT1a group undergoing surgical resection of IPMN (Graph 2). **(C)**. Confusion matrix of the decision tree model **(A)** and the random forest model **(B)** on the testing set. The confusion matrix of on the testing set.

In the training set, 41 (13%) patients died with a median follow-up time of 99 months (IQR, 2–120). The estimated overall 5-year survival rate was 95.1% for patients with IPMN with LGD, 61.5% for patients with HGD, 55.0% for patients with pT1a, and 40.6% for patients with I-IPMC beyond pT1a. There was no difference in median OS between HGD IPMN and pT1a IPMC (65.39 vs. 68.62 months, P = 0.178) (**Figure 3A**). So, HGD and pT1a were integrated into the HGD/pT1a group for analysis. When the median OS of HGD/pT1a IPMN was compared with that of LGD IPMN, there was a marked significant difference (71.55 vs. 108.87 months, P = 0.000,). There was also a marked significant difference when the median OS of HGD/pT1a IPMN was compared with that of I-IPMC beyond pT1a (71.55 vs. 50.57 months, P = 0.005) (**Figure 3B**).

### Factors associated with subtypes of IPMN in the training set in univariate analyses

The variables included in the univariate analysis are shown in **Table 2**. For IPMN, HGD, pT1a, and invasive malignancy beyond pT1a were more prevalent in patients who presented with mixed type, presence of mural nodules, MPD, and common bile duct dilatation than LGD IPMN whereas LGD IPMN was more common with branch duct type (P < 0.05). I-IPMC beyond the pT1a group was significantly more prevalent when presented with concomitant new-onset diabetes mellitus (DM), unclear focus boundary, elevated serum CA19-9, and obstructive jaundice as compared with LGD, HGD, and pT1a IPMN groups for which there was no statistical difference among LGD, HGD and pT1a IPMN groups. Our study showed no significant differences in all variables between patients with HGD IPMN and pT1a IPMC, except for distal pancreatic atrophy which was more common in pT1a IPMC.

**Table 2:** Univariate analysis of preoperative (clinical or radiological) findings associated with subtypes of IPMNs in the training set.

Pathological Diagnosis	(1)Patients With LGD IPMN (n=139)	(2)Patients With HGD IPMN (n=13)	(3) Patients With p1Ta IPMN (n=24)	(4)Patients With I-IPMC beyond pT1a (n=34)	P Value
Mean age (range), yrs	60.3 (80,27)a	66.0 (70,56)	64.5 (76,41)	65.3 (84,53)a	0.014
Male %	80 (57.6%)	5 (38.5%)	16 (66.7%)	16 (47.1%)	0.266
BMI,kg/m2 (range)	22.3 (30.0,14.4)	21.3 (24.6,17.3)	21.8 (26.3,16.4)	21.6 (27.2,15.2)	0.466
Initial symptoms					0.01
Jaundice	2 (1.4%)	0 (0.0%)	0 (0.0%)	2 (5.9%)	
Weight loss	5 (3.6%)	0 (0.0%)	3 (12.5%)	2 (5.9%)	
Abdominal pain	69 (49.6%)	10 (76.9%)d	9 (37.5%)d	20 (58.8%)	
Back pain	4 (2.9%)	0 (0.0%)	3 (12.5%)	4 (11.8%)	
No chief complaints	59 (42.4%)a	3 (23.1%)	11 (45.8%)	6 (17.6%)a	
Duration of symptoms, months	10.8 (120,0.1)	8.8 (60.0,0.3)	4.9 (30,0.3)	3.7 (24,0.3)	0.11
Family history of pancreatic cancer	6 (4.3%)	0 (0.0%)	1 (4.2%)	1 (2.9%)	0.875
Diabetes	37 (26.6%)a	2 (15.4%)	8 (33.4%)	18 (53.0%)a	0.005
New-onset DM	23 (16.5%)a	1 (7.7%)	4 (16.7%)	16 (47.1%)a	
Long-standing DM	14 (10.1%)	1 (7.7%)	4 (16.7%)	2 (5.9%)	
HBV	14 (10.1%)	2 (5.4%)	0 (0.0%)	3 (8.8%)	0.362
Acute pancreatitis	22 (15.8%)	0 (0.0%)	5 (20.8%)	6 (18.2%)	0.386
tumor location					0.003
Non diffuse	128 (92.1%)	12 (92.3%)	15 (62.5%)	29 (85.3%)	
Head	78 (56.1%)	7 (53.8%)	11 (45.8%)	22 (64.7%)	
Body/tail	50 (36.0%)	5 (38.5%)	4 (16.7%)	7 (20.6%)	
Diffuse	11 (7.9%)a	1 (7.7%)	9 (37.5%)	5 (14.7%)a	
Morphological types on imaging findings					<0.001
Main duct/Mixed	45 (32.4%)	11 (84.6%)	21 (87.5%)	22 (64.7%)	
Main duct	14 (10.1%)	0 (0.0%)	5 (20.8%)	8 (23.5%)	
Mixed	31 (22.3%)bf	11 (84.6%)f	16 (66.7%)b	14 (41.2%)	
Branch duct	94 (67.6%)abf	2 (15.4%)f	3 (12.5%)b	12 (35.3%)a	
Maximum size, median (range), cm	2.8 (19.6,1.0)b	3.6 (5.8,0.7)	4.0 (8.5,1.2)b	3.2 (8.8,1.73)	0.027
Presence of mural nodule, %	32 ( 23.0%)abf	10 (76.9%)f	21 (87.5%)b	30 (88.2%)a	<0.001
Maximum size of mural nodule, median (range), mm	2.0 (4.3,0.0)abf	0.9 (1.9,0.0)cf	1.1 (3.5,0.0)be	1.6 (4.5,0.0)ace	<0.001
Lesion edge Clear,%	120 (86.3%) ab	10 (76.9%)c	11 (45.8%)b	5 (14.7%)ac	<0.001
Maximum diameter of main pancreatic duct, median (range), cm	0.4 (2.9,0.1)abf	1.1 (4.3,0.4)f	1.3 (3.9,0.5)be	0.84 (3.0,0.2)ae	<0.001
CBD dilatation,%	1 (0.7%) ab	1 (7.7%)c	9 (37.5%)b	17 (50%)ac	<0.001
Distal pancreatic atrophy,%	25 (18.0%)ab	4 (30.8%)d	20 (83.3%)bd	22 (64.7%)a	<0.001
Serum CA19-9, U/mL	28.0 (1141.9,0.2)a	20.3 (136.0,3.3)c	63.6 (460.4,5.0)e	348.8 (4446.2,1.1)ace	<0.001
Serum CEA, ng/Ml	2.6 (17.1,0.6)a	4.2 (20.4,1.0)	4.8 (15.9,0.7)	7.4 (96.0,1.2)a	0.006
Serum CA125, U/mL	10.3 (62.8,2.0)a	16.8 (70.6,7.7)	14.9 (41.1,4.2)	21.2 (269.9,4.4)a	0.029
Serum AFP, ng/mL	3.5 (41.9,0.8)	3.4 (6,1.2)	3.2 (7.9,1.2)	3.3 (24.0,1.0)	0.963
Liver function					<0.001
Obstructive jaundice	1 (0.7%)a	0 (0.0%)	2 (14.3%)	11 (32.4%)a	

Categorical values are expressed as percentages, and P values calculated with Chi square analysis. Continuous variables are shown as median and interquartile ranges and compared with ANOVA. P-values denote comparisons among the four groups. ab and cdef signify P < 0.05 for pairwise comparisons. a:1-4, b:1-3, c:2-4, d:2-3, e:3-4, and f:1-2.

BMI, body mass index; DM, diabetes mellitus; HBV, hepatitis B virus; CBD, common bile duct; CA19-9, carbohydrate antigen 19-9; CEA, carcinoembryonic antigen; CA125, carbohydrate antigen 125; AFP, alpha-fetoprotein; LGD, low grade dysplasia; HGD, high grade dysplasia; p1Ta, invasive component ≤ 5 mm; IPMN, intraductal papillary mucinous neoplasm; I-IPMC, invasive intraductal papillary mucinous carcinoma; NA, not applicable.

### The 3-class classification results of training and testing sets including LGD, HGD/pT1a IPMN and IPMC beyond pT1a

In the decision tree model, we used cross entropy as the information gain and set the maximum depth of the tree as 5. In the training set, an accuracy of 92.4% was reported (194 of 210 correct), and with the testing set, an accuracy of 86.9% (86 of 99 correct). The confusion matrix of the decision tree model on the testing set is shown in **Figure 3C**. On the decision tree model, we have made an internal validation on our own dataset by a 10-fold cross validation experiment, and an average accuracy of 78.1% is reported.

In the random forest model, we used cross entropy as the information gain and set the number of trees as 30, and the random features chosen for each tree is set as the square root of the total number of features. The random forest model has an accuracy of 99.5% (209 of 210 correct) and 96.0% with the testing set (95 of 99 correct). The confusion matrix of the random forest model on the testing set is also shown in **Figure 3C**. An internal cross validation is also conducted on the random forest model, and the average accuracy is 90.6%.

**Figure 4:**
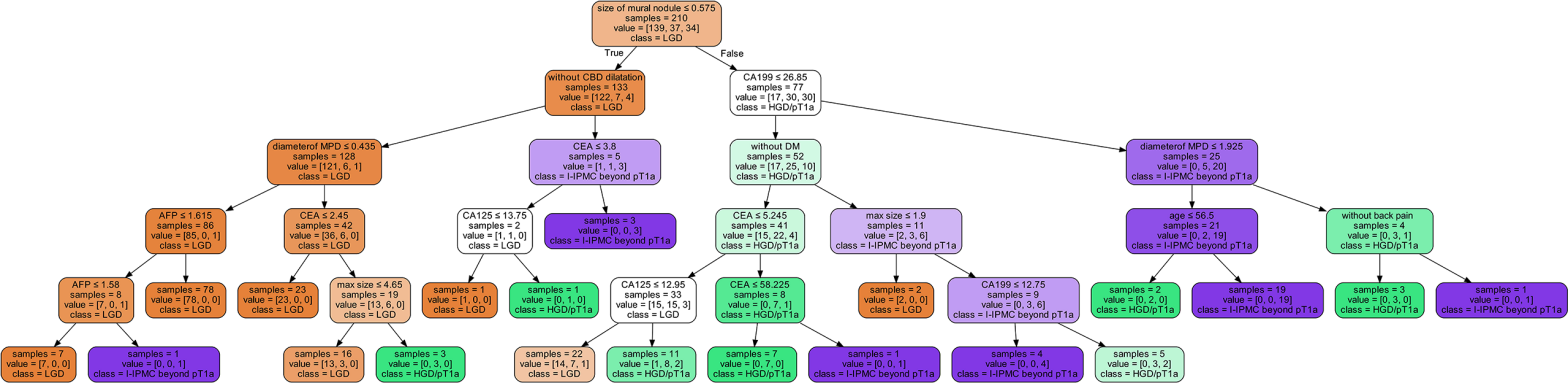
Illustration of the decision tree model.

**Figure 5:**
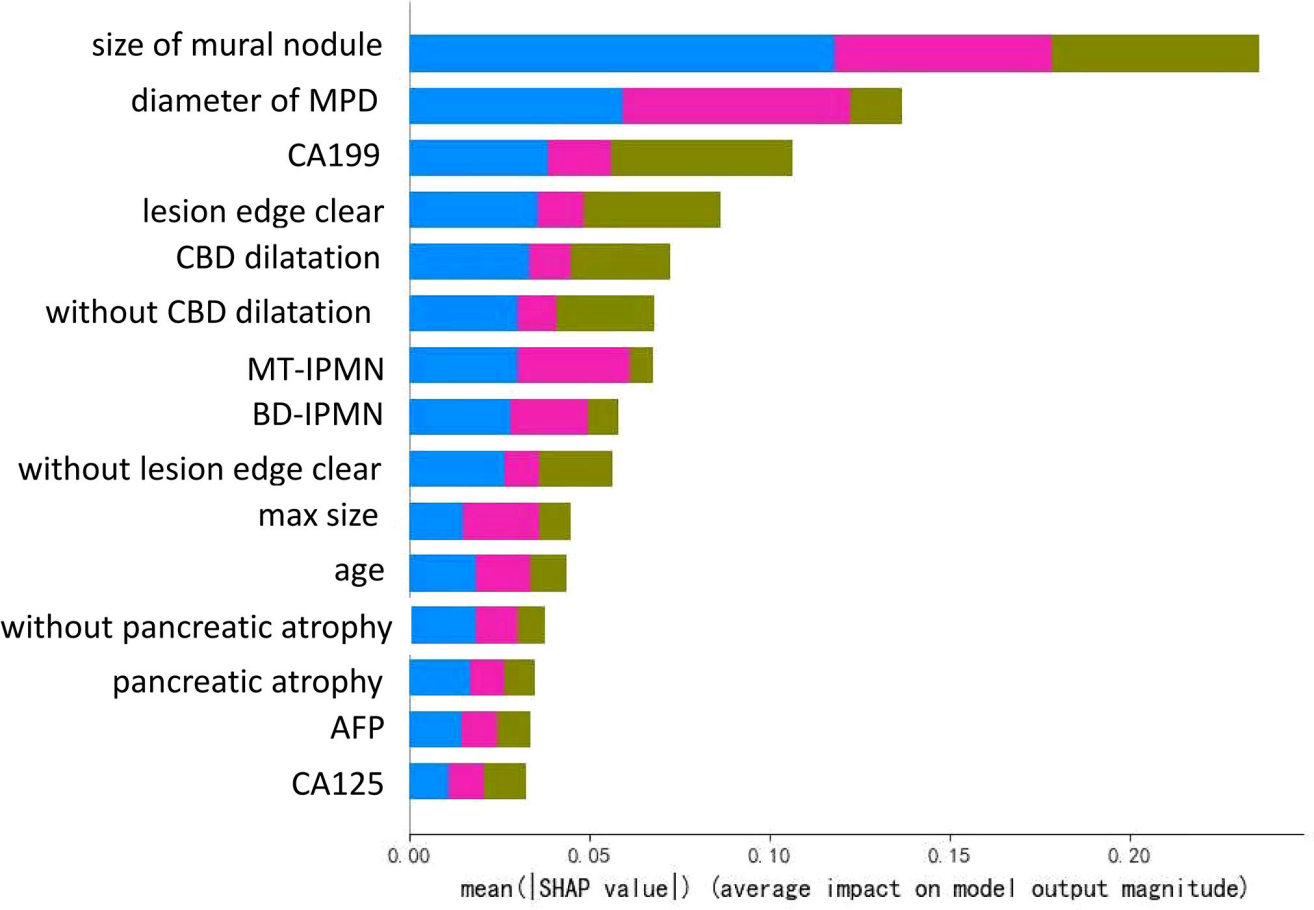
Illustration of the interpretation model of the decision tree. The bar lengths represent the mean |SHAP value|, which shows the significance of a single factor in the classification model. Different colors show the significance of different classes, where the blue, green, and pink bars represent the mean |SHAP value| for the categories LGD IPMN, HGD/pT1a IPMN, and I-IPMC beyond pT1a, respectively.

The decision tree model has strong interpretation; our trained decision tree model is shown in **Figure 4**. The nodes at the top part of the trees, as the mural nodule size, CBD dilation, and serum CA19-9 levels, were the most important factors of the model. For interpretability of the random forest model with respect to the input factors, we used the estimated SHAP value, which is shown in **Figure 5**. The blue, green, and pink bars represent the mean |SHAP value| for the classes LGD IPMN, HGD/pT1a IPMN, and I-IPMC beyond pT1a, respectively. Longer bars imply stronger significance, and only the top 15 factors in order are shown. Among them, the mural nodule size, MPD diameter, serum CA19-9 levels, lesion edge clear, and CBD dilation were the most important factors that influenced the classification results in the interpretation of the random forest model. There were some discrepancies in the range of the importance of factors for the random forest model between the decision tree model, which a higher accuracy indicated a more comprehensive interpretation than a single decision tree.

## Discussion

The current consensus regarding the malignant potential of IPMN is that its aggressiveness is dependent on the presence of invasive cancer, the extent of cancer invasion, and the biological characteristics of cancer cells (24–30). These findings suggest that the therapeutic strategy for IPMN should differ according to the grade. Therefore, it is becoming more important to accurately categorize IPMN preoperatively into its corresponding pathological subtypes. Previous studies have focused on I-IPMC, but did not focus on the early stage of malignant IPMN. However, in the clinic, such patients are the most difficult to identify in time, and if identified earlier, the prognosis of patients will be greatly improved. In our study, HGD IPMN shows similar optimistic postoperative outcome to pT1a IPMC, whereas I-IPMC beyond pT1a has a poorer outcome compared with HGD/pT1a IPMN. The postoperative survival and 5-year survival rate of IPMN were nearly consistent with previous reports (22, 45). Moreover, there were no significant differences between most variables between HGD IPMN and pT1a IPMC in the present study. Therefore, we believe that pT1a and HGD behave similarly and both have excellent prognoses; thus, we integrated both pT1a and HGD into HGD/pT1a IPMN in the present study. However, performing grade classification preoperatively is generally difficult, and preoperative discrimination between IPMN with HGD/pT1a and LGD is nearly impossible. Several predictive factors for malignant IPMN have been reported, including dilated branch (30 mm), MPD dilatation, the presence of mural nodules, elevated CEA, and elevated CA19-9. These factors, however, were not able to differentiate HGD/pT1a from LGD lesions. Similarly, Kang et al. found that adding diffusion-weighted images to MRCP improved the ability to detect invasive malignancy in IPMN (46). In a recent study, the authors found that the use of contrast-enhanced harmonic EUS (CH-EUS) provided an increased diagnostic yield in the identification of malignant features of mural nodules within pancreatic cystic neoplasms (PCNs) (47). However, defining the degree of dysplasia in IPMN was not possible with imaging and classical tumor markers or EUS characteristics alone. In this study, we focused on establishing a pioneering system that combines the features into a patient-specific risk using unique clinical and standardized preoperative examination modalities and standardized pathological diagnostic criteria by tree models to perform accurate grade classification preoperatively.

The present study found 16 variables to be independently associated with the progression from LGD to HGD and pT1a to invasive carcinoma (**Table 2**). Among them, accompanied by an unclear focus boundary, DM (especially new-onset DM), elevated serum CA19-9, and obstructive jaundice are obvious in the advanced stage of invasive IPMC, but are not common in HGD or pT1a IPMC. Many previous studies and guidelines have suggested that elevated serum CA19-9, DM, and obstructive jaundice are risk predictors of malignant IPMN (18, 20, 28, 45, 48, 49). However, HGD and pT1a IPMN have not been previously analyzed separately from malignant IPMN. Thus, in the present study, we found the three factors could not be used as a sign of early malignancy, and their appearance often indicates that the disease is at an advanced stage of invasion. Moreover, our study suggests the presence of mural nodules should be regarded as highly suspicious of invasive carcinoma or HGD in all morphological types, which is consistent with a malignant predictor in several reports (32, 43, 48, 50, 51). However, we also found that nearly 12–23% of cases of IPMN in the HGD and I-IPMC groups had no mural nodules; thus, other factors need to be considered. CBD diameter ≥ 15 mm and MPD dilatation are two specific signs of HGD and invasive disease (LGD *vs*. HGD *vs*. pT1a *vs*. I-IPMC beyond pT1a, 1 [0.7%] *vs*. 1 [7.7%] *vs*. 9 [37.5%] *vs*. 17[50.0%], p = 0.000; 0.4 (2.9, 0.1) *vs*. 1.1 (4.3, 0.4) *vs*. 1.3 (3.9, 0.5) *vs*. 0.84 (3.0, 0.2), p = 0.000). Although MT-IPMN was also associated with malignant disease (P = 0.000), the pathology of 35.3% of BD-IPMN was I-IPMC beyond pT1a. Assessing the malignant risk of BD-IPMN will require further investigation.

To observe the influence of high-risk factors, we used the interpretability method for tree models, which can help improve clinician understanding of IPMNs and important characteristics for diagnosis. As shown in **Figure 4**, the nearest nodes to the root of the decision tree have the greatest influence on the classification. For a simple analysis, we can see the key to distinguishing benign from malignant IPMN is mural nodules. If the mural nodule size is less than 0.575 cm, LGD IPMN is more likely. If the mural nodule size is greater than 0.575 cm, and CA19-9 is greater than 26.85 U/ml, IPMC is more likely. If the mural nodule size is greater than 0.575 cm, but CA19-9 is less than 26.85 U/ml, no history of diabetes, and CEA is greater than 5.245 ng/ml, HGD/pT1a is more likely; if CEA is less than 5.245 ng/ml, LGD is more likely. From this, we can see the appearance of mural nodules( >0.575 cm) suggests early or advanced stage malignant IPMC and elevated serum CA19-9 reflects an advanced stage of invasive IPMC, which is similar to the recently published paper (52). In addition, the mural nodules of our decision tree algorithm was 5.75mm, and it can also be considered as a cutoff value, which was closed to previous studies (4, 43, 53),and the high accuracy of our model showed unique practical significance in our classification of LGD, HGD/pT1a and I-IPMC beyond pT1a. In the random forest model, which had a higher accuracy, the SHAP value was used for interpretation. As in **Figure 5**, the mural nodule size, MPD diameter, serum CA19-9 levels, whether the lesion edge was clear, and the presence of CBD dilatation all influenced the random forest classification model. The decision tree provided an outstanding prediction capability for each stage for IPMN, with an accuracy of 86.9% on the testing set. The random forest model also showed high diagnostic ability with an accuracy of 96.0% on the testing set.

The random forest model uses the ensemble learning method, which assembles multiple decision trees, and as a result, is more accurate for this 3-class classification task. Comprehensive classification, including benign, early malignant, and advanced invasive, is convenient for clinical decisions. Additionally, we have made a convenient software for this random forest model for clinicians. By inputting relative captured features, the predicted class of IPMN of a single patient can be shown.

There are some limitations to the present study. First, this model was developed in a population of patients who were subsequently treated surgically. Hence, the cohort may not accurately represent patients with the whole IPMN population, and the cases could have selection bias. Second, prospective investigations conducted at multiple institutions are necessary for validating the results obtained in the current study. Finally, HGD and pT1a IPMN patients were relatively few. Therefore, internal validation (10-fold cross-validation) was used to evaluate the diagnostic performance for the invasion potency of IPMN because we could not collect enough patients to perform AI when patients were separated into groups for training and test data, and the results of the cross-validation have also shown high accuracy in classifying LGD, HGD/pT1a, and I-IPMC beyond pT1a, which better proves the efficiency of our AI models.

Overall, a simple and objective predictive model for IPMN of each stage that can be used for any lesion type was constructed. This predictive model provides important information for clinicians and patients in assessing an individual’s risk for early stage malignant IPMN and may help identify masses that appear benign but require surgery.

## Data availability statement

The raw data supporting the conclusions of this article will be made available by the authors, without undue reservation.

## Ethics statement

The studies involving human participants were reviewed and approved by Ruijin hospital Ethics Committee Shanghai Jiaotong University School of Medicine. Written informed consent for participation was not required for this study in accordance with the national legislation and the institutional requirements.

## Author contributions

XH, DZ and YY had full access to all the data in the study and take responsibility for the integrity of the data and the accuracy of the data analysis. Concept and design : XH, RF, DZ. Acquisition, analysis, or interpretation of data: XH, RF, JS, YR. Drafting of the manuscript: XH. Critical revision of the manuscript for important intellectual content: DZ and YY. Statistical analysis: XH. Technical, or material support: XZ, WR. All authors contributed to the article and approved the submitted version.

## References

[1] Brosens LA Hackeng WM Offerhaus GJ Hruban RH Wood LD. Pancreatic adenocarcinoma pathology: Changing "landscape". J Gastrointest Oncol (2015) 6:358–74. doi: 10.3978/j.issn.2078-6891.2015.032 PMC4502163 26261723

[2] Nagtegaal ID Odze RD Klimstra D Paradis V Rugge M Schirmacher P. The 2019 WHO classification of tumours of the digestive system. Histopathology (2020) 76:182–88. doi: 10.1111/his.13975 PMC7003895 31433515

[3] McMillan MT Lewis RS Drebin JA Teitelbaum UR Lee MK Roses RE. The efficacy of adjuvant therapy for pancreatic invasive intraductal papillary mucinous neoplasm (IPMN). Cancer (2016) 122:521–33. doi: 10.1002/cncr.29803 26587698

[4] Kang MJ Jang JY Lee KB Chang YR Kwon W Kim SW. Long-term prospective cohort study of patients undergoing pancreatectomy for intraductal papillary mucinous neoplasm of the pancreas: Implications for postoperative surveillance. Ann Surg (2014) 260:356–63. doi: 10.1097/SLA.0000000000000470 24378847

[5] Hirono S Tani M Kawai M Ina S Nishioka R Miyazawa M. Treatment strategy for intraductal papillary mucinous neoplasm of the pancreas based on malignant predictive factors. Arch Surg (2009) 144:345–49. discussion349-50. doi: 10.1001/archsurg.2009.2 19380648

[6] Winter JM Jiang W Basturk O Kenudson MM Fong ZV Tan WP. Recurrence and survival after resection of small intraductal papillary mucinous neoplasm-associated carcinomas (</=20-mm invasive component): A multi-institutional analysis. Ann Surg (2016) 263:793–801. doi: 10.1097/SLA.0000000000001319 26135696 PMC4957241

[7] Wasif N Bentrem DJ Farrell JJ Ko CY Hines OJ Reber HA. Invasive intraductal papillary mucinous neoplasm versus sporadic pancreatic adenocarcinoma: a stage-matched comparison of outcomes. Cancer (2010) 116:3369–77. doi: 10.1002/cncr.25070 PMC2902567 20564064

[8] Yopp AC Katabi N Janakos M Klimstra DS D'Angelica MI DeMatteo RP. Invasive carcinoma arising in intraductal papillary mucinous neoplasms of the pancreas: A matched control study with conventional pancreatic ductal adenocarcinoma. Ann Surg (2011) 253:968–74. doi: 10.1097/SLA.0b013e318214bcb4 21422912

[9] Poultsides GA Reddy S Cameron JL Hruban RH Pawlik TM Ahuja N. Histopathologic basis for the favorable survival after resection of intraductal papillary mucinous neoplasm-associated invasive adenocarcinoma of the pancreas. Ann Surg (2010) 251:470–76. doi: 10.1097/SLA.0b013e3181cf8a19 PMC3437748 20142731

[10] Shimizu Y Hijioka S Hirono S Kin T Ohtsuka T Kanno A. New model for predicting malignancy in patients with intraductal papillary mucinous neoplasm. Ann Surg (2020) 272:155–62. doi: 10.1097/SLA.0000000000003108 30499803

[11] Sahora K Mino-Kenudson M Brugge W Thayer SP Ferrone CR Sahani D. Branch duct intraductal papillary mucinous neoplasms: does cyst size change the tip of the scale? a critical analysis of the revised international consensus guidelines in a large single-institutional series. Ann Surg (2013) 258:466–75. doi: 10.1097/SLA.0b013e3182a18f48 24022439

[12] Fritz S Klauss M Bergmann F Strobel O Schneider L Werner J. Pancreatic main-duct involvement in branch-duct IPMNs: an underestimated risk. Ann Surg (2014) 260:848–55. discussion 855-46. doi: 10.1097/SLA.0000000000000980 25379856

[13] Aso T Ohtsuka T Matsunaga T Kimura H Watanabe Y Tamura K. "High-risk stigmata" of the 2012 international consensus guidelines correlate with the malignant grade of branch duct intraductal papillary mucinous neoplasms of the pancreas. Pancreas (2014) 43:1239–43. doi: 10.1097/MPA.0000000000000199 25036910

[14] Roch AM Ceppa EP Al-Haddad MA DeWitt JM House MG Zyromski NJ. The natural history of main duct-involved, mixed-type intraductal papillary mucinous neoplasm: parameters predictive of progression. Ann Surg (2014) 260:680–8. discussion 688-90. doi: 10.1097/SLA.0000000000000927 25203885

[15] Goh BK Thng CH Tan DM Low AS Wong JS Cheow PC. Evaluation of the Sendai and 2012 international consensus guidelines based on cross-sectional imaging findings performed for the initial triage of mucinous cystic lesions of the pancreas: a single institution experience with 114 surgically treated patients. Am J Surg (2014) 208:202–9. doi: 10.1016/j.amjsurg.2013.09.031 24530043

[16] Jang JY Park T Lee S Kang MJ Lee SY Lee KB. Validation of international consensus guidelines for the resection of branch duct-type intraductal papillary mucinous neoplasms. Br J Surg (2014) 101:686–92. doi: 10.1002/bjs.9491 24668442

[17] Robles EP Maire F Cros J Vullierme MP Rebours V Sauvanet A. Accuracy of 2012 international consensus guidelines for the prediction of malignancy of branch-duct intraductal papillary mucinous neoplasms of the pancreas. United Eur Gastroenterol J (2016) 4:580–6. doi: 10.1177/2050640615623370 PMC4971792 27536368

[18] Tanaka M Fernandez-del Castillo C Adsay V Chari S Falconi M Jang JY. International consensus guidelines 2012 for the management of IPMN and MCN of the pancreas. Pancreatology (2012) 12:183–97. doi: 10.1016/j.pan.2012.04.004 22687371

[19] Tanaka M Chari S Adsay V Castillo CF Falconi M Shimizu M. International consensus guidelines for management of intraductal papillary mucinous neoplasms and mucinous cystic neoplasms of the pancreas. Pancreatology (2006) 6:17–32. doi: 10.1159/000090023 16327281

[20] European Study Group on Cystic Tumours of the Pancreas. European Evidence-based guidelines on pancreatic cystic neoplasms. Gut (2018) 67:789–804. doi: 10.1136/gutjnl-2018-316027 29574408 PMC5890653

[21] Tanaka M Fernandez-Del Castillo C Kamisawa T Jang JY Levy P Ohtsuka T. Revisions of international consensus Fukuoka guidelines for the management of IPMN of the pancreas. Pancreatology (2017) 17:738–53. doi: 10.1016/j.pan.2017.07.007 28735806

[22] Rezaee N Barbon C Zaki A He J Salman B Hruban RH. Intraductal papillary mucinous neoplasm (IPMN) with high-grade dysplasia is a risk factor for the subsequent development of pancreatic ductal adenocarcinoma. HPB (Oxf) (2016) 18:236–46. doi: 10.1016/j.hpb.2015.10.010 PMC4814593 27017163

[23] Schnelldorfer T Sarr MG Nagorney DM Zhang LZ Smyrk TC Qin R. Experience with 208 resections for intraductal papillary mucinous neoplasm of the pancreas. Arch Surg (2008) 143:639–46. discussion 646. doi: 10.1001/archsurg.143.7.639 18645105

[24] Chari ST Yadav D Smyrk TC DiMagno EP Miller LJ Raimondo M. Study of recurrence after surgical resection of intraductal papillary mucinous neoplasm of the pancreas. Gastroenterology (2002) 123:1500–7. doi: 10.1053/gast.2002.36552 12404225

[25] D'Angelica M Brennan MF Suriawinata AA Klimstra D Conlon KC. Intraductal papillary mucinous neoplasms of the pancreas: an analysis of clinicopathologic features and outcome. Ann Surg (2004) 239:400–8. doi: 10.1097/01.sla.0000114132.47816.dd PMC1356240 15075659

[26] Maire F Hammel P Terris B Paye F Scoazec JY Cellier C. Prognosis of malignant intraductal papillary mucinous tumours of the pancreas after surgical resection. comparison with pancreatic ductal adenocarcinoma. Gut. (2002) 51:717–22. doi: 10.1136/gut.51.5.717 PMC1773420 12377813

[27] Raimondo M Tachibana I Urrutia R Burgart LJ DiMagno EP. Invasive cancer and survival of intraductal papillary mucinous tumors of the pancreas. Am J Gastroenterol (2002) 97:2553–8. doi: 10.1111/j.1572-0241.2002.06022.x 12385438

[28] Salvia R Fernandez-del Castillo C Bassi C Thayer SP Falconi M Mantovani W. Main-duct intraductal papillary mucinous neoplasms of the pancreas: clinical predictors of malignancy and long-term survival following resection. Ann Surg (2004) 239:678–85. discussion 685-77. doi: 10.1097/01.sla.0000124386.54496.15 PMC1356276 15082972

[29] Shimada K Sakamoto Y Sano T Kosuge T Hiraoka N. Invasive carcinoma originating in an intraductal papillary mucinous neoplasm of the pancreas: A clinicopathologic comparison with a common type of invasive ductal carcinoma. Pancreas. (2006) 32:281–7. doi: 10.1097/01.mpa.0000202955.33483.e2 16628084

[30] Sohn TA Yeo CJ Cameron JL Hruban RH Fukushima N Campbell KA. Intraductal papillary mucinous neoplasms of the pancreas: an updated experience. Ann Surg (2004) 239:788–97. discussion 797-8. doi: 10.1097/01.sla.0000128306.90650.aa PMC1356287 15166958

[31] Sadakari Y Ienaga J Kobayashi K Miyasaka Y Takahata S Nakamura M. Cyst size indicates malignant transformation in branch duct intraductal papillary mucinous neoplasm of the pancreas without mural nodules. Pancreas (2010) 39:232–6. doi: 10.1097/MPA.0b013e3181bab60e 19752768

[32] Shimizu Y Yamaue H Maguchi H Yamao K Hirono S Osanai M. Predictors of malignancy in intraductal papillary mucinous neoplasm of the pancreas: analysis of 310 pancreatic resection patients at multiple high-volume centers. Pancreas (2013) 42:883–8. doi: 10.1097/MPA.0b013e31827a7b84 23508017

[33] Scott ML Su-In L. (2017). A unified approach to interpreting model predictions, in: NIPS'17: Proceedings of the 31st International Conference on Neural Information Processing Systems. pp. 4768–77.

[34] Yadav RK Jiang X Chen J. Differentiating benign from malignant pancreatic cysts on computed tomography. Eur J Radiol Open (2020) 7:100278. a. doi: 10.1016/j.ejro.2020.100278 33163586 PMC7607418

[35] Hirooka Y Goto H Itoh A Hashimoto S Niwa K Ishikawa H. Case of intraductal papillary mucinous tumor in whichendosonography-guided fine-needle aspiration biopsy caused dissemination. J Gastroenterol Hepatol (2003) 18:1323–24. doi: 10.1046/j.1440-1746.2003.03040.x 14535994

[36] Katanuma A Maguchi H Hashigo S Kaneko M Kin T Yane K. Tumor seeding after endoscopic ultrasound-guided fine-needle aspiration of cancer in the body of the pancreas. Endoscopy (2012) 44(suppl 2, UCTN):E160–1. doi: 10.1055/s-0031-1291716 22622721

[37] Paquin SC Gariépy G Lepanto L Bourdages R Raymond G Sahai AV. A first report of tumor seeding because of EUS-guided FNA of a pancreatic adenocarcinoma. Gastrointest Endosc (2005) 61:610–11. doi: 10.1016/S0016-5107(05)00082-9 15812422

[38] Facciorusso A Kovacevic B Yang D Boas FV Moreno BM Stigliano S. Predictors of adverse events after endoscopic ultrasound-guided through-the-needle biopsy of pancreatic cysts: A recursive partitioning analysis. Endoscopy (2022) 54(12):1158–68. doi: 10.1055/a-1831-5385 35451041

[39] Wang D Zhou J Zheng S Xia J Hu H. Evaluation of intraductal papillary mucinous neoplasms of the pancreas on MDCT and MRI. Zhonghua Zhong Liu Za Zhi (2014) 36:682–7. 25564059

[40] Kim MJ Mitchell DG Ito K Outwater EK. Biliary dilatation: differentiation of benign from malignant causes-value of adding conventional MR imaging to MR cholangiopancreatography. Radiology (2000) 214:173–81. doi: 10.1148/radiology.214.1.r00ja35173 10644119

[41] Kang MJ Jang JY Lee S Park T Lee SY Kim SW. Clinicopathological meaning of size of main-duct dilatation in intraductal papillary mucinous neoplasm of pancreas: Proposal of a simplified morphological classification based on the investigation on the size of main pancreatic duct. World J Surg (2015) 39:2006–13. doi: 10.1007/s00268-015-3062-0 25894399

[42] Kwong WT Lawson RD Hunt G Fehmi SM Proudfoot JA Xu R. Rapid growth rates of suspected pancreatic cyst branch duct intraductal papillary mucinous neoplasms predict malignancy. Dig Dis Sci (2015) 60:2800–6. doi: 10.1007/s10620-015-3679-8 25924899

[43] Hirono S Tani M Kawai M Okada K Miyazawa M Shimizu A. The carcinoembryonic antigen level in pancreatic juice and mural nodule size are predictors of malignancy for branch duct type intraductal papillary mucinous neoplasms of the pancreas. Ann Surg (2012) 255:517–22. doi: 10.1097/SLA.0b013e3182444231 22301608

[44] Sobin LH Compton CC. TNM seventh edition: what's new, what's changed: communication from the international union against cancer and the American joint committee on cancer. Cancer (2010) 116(22):5336–9. doi: 10.1002/cncr.25537 20665503

[45] Nara S Shimada K Kosuge T Kanai Y Hiraoka N. Minimally invasive intraductal papillary-mucinous carcinoma of the pancreas: clinicopathologic study of 104 intraductal papillary-mucinous neoplasms. Am J Surg Pathol (2008) 32:243–55. doi: 10.1097/PAS.0b013e3181484f1e 18223327

[46] Kang KM Lee JM Shin CI Baek JH Kim SH Yoon JH. Added value of diffusion-weighted imaging to MR cholangiopancreatography with unenhanced mr imaging for predicting malignancy or invasiveness of intraductal papillary mucinous neoplasm of the pancreas. J Magn Reson Imag. (2013) 38:555–63. doi: 10.1002/jmri.24022 23390008

[47] Lisotti A Napoleon B Facciorusso A Cominardi A Crinò SF Brighi N. Contrast-enhanced EUS for the characterization of mural nodules within pancreatic cystic neoplasms: Systematic review and meta-analysis. Gastrointest Endosc (2021) 94(5):881–9. doi: 10.1016/j.gie.2021.06.028 34217751

[48] Kim KW Park SH Pyo J Yoon SH Byun JH Lee MG. Imaging features to distinguish malignant and benign branch-duct type intraductal papillary mucinous neoplasms of the pancreas: A meta-analysis. Ann Surg (2014) 259:72–81. doi: 10.1097/SLA.0b013e31829385f7 23657084

[49] Duconseil P Adham M Sauvanet A Autret A Périnel J Chiche L. Fukuoka-Negative branch-duct IPMNs: When to worry? a study from the French surgical association (AFC). Ann Surg Oncol (2018) 25:1017–25. doi: 10.1245/s10434-017-6318-0 29392508

[50] Seo N Byun JH Kim JH Kim HJ Lee SS Song KB. Validation of the 2012 international consensus guidelines using computed tomography and magnetic resonance imaging: Branch duct and main duct intraductal papillary mucinous neoplasms of the pancreas. Ann Surg (2016) 263:557–64. doi: 10.1097/SLA.0000000000001217 25822687

[51] Moris M Raimondo M Woodward TA Skinner V Arcidiacono PG Petrone MC. Risk factors for malignant progression of intraductal papillary mucinous neoplasms. Dig Liver Dis (2015) 47:495–501. doi: 10.1016/j.dld.2015.03.007 25869552

[52] Xu Y Xie C Gao Z Zhang M Zhan M. Nomogram to predict malignancy in branch duct type intraductal papillary mucinous neoplasms. Med (Baltimore). (2022) 101(38):e30627. doi: 10.1097/MD.0000000000030627 PMC9509101 36197166

[53] Anand N Sampath K Wu BU. Cyst features and risk of malignancy in intraductal papillary mucinous neoplasms of the pancreas: a metaanalysis. Clin Gastroenterol Hepatol (2013) 11:913–21. doi: 10.1016/j.cgh.2013.02.010 23416279

